# Insights into Molecular and Cellular Mechanisms of NeuroCOVID

**DOI:** 10.3390/cells13211790

**Published:** 2024-10-29

**Authors:** Christian Barbato, Carla Petrella, Antonio Minni

**Affiliations:** 1 Institute of Biochemistry and Cell Biology (IBBC), National Research Council (CNR), Via E. Ramarini, 32, Monterotondo Scalo, 00015 Roma, Italy; carla.petrella@cnr.it; 2 Department of Sense Organs DOS, Sapienza University of Rome, Viale del Policlinico 155, 00161 Roma, Italy; antonio.minni@uniroma1.it; 3 Division of Otorhinolaryngology-Head and Neck Surgery, Ospedale San Camillo de Lellis, ASL Rieti-Sapienza University, Viale Kennedy, 02100 Rieti, Italy

Post-COVID-19 syndrome (PCS) is defined by the persistence or recurrence of symptoms after an initial acute SARS-CoV-2 infection. This condition continues with symptoms that last for at least 2 months, usually 3 months from the onset of COVID-19. It cannot be attributed to other clinical situations or diagnoses, and its persistence was collectively termed long COVID [1]. According to a recent estimate, long COVID is a disease experienced by as many as 400 million people worldwide [2]. PCS patients are affected by neurological, cardiac, and respiratory symptoms like dyspnea, fatigue, loss of olfactory and taste function with anosmia and dysgeusia, neurocognitive symptoms defined as “brain fog”, sleep disturbance, weakness, and psychological impairment reducing physical and mental quality of life. In particular, the involvement of the brain, and more generally the central and peripheral nervous systems, as the potential target organs of SARS-CoV-2 that can be affected during and after infection, has been defined as neuroCOVID. Numerous studies have depicted neurological and neuropsychological complications, and the effects of COVID-19 on the nervous system are becoming sharply defined [3]. Now, it is evident that these disorders can also affect young people (30–50 years of age) who (1) had never previously experienced these symptoms, (2) had developed even very mild forms of COVID-19, or (3) had not experienced breathing problems.

This Special Issue “Insights into Molecular and Cellular Mechanisms of NeuroCOVID” linked together new research and review articles that expand our understanding of the molecular and cellular mechanisms of neuroCOVID disease. Contributions focus on clinical outcomes, offering interesting insights into current understanding and identifying the following future directions:

- Exploring integrated models of translational biomedical research starting from long-lasting post-infective neurobiological modifications, (planned Special Issue 2nd edition).
- Exploring common molecular and cellular determinants of the neurosensorial and neurological clinical manifestation of neuroCOVID wave [4].
- Providing recent molecular and cellular neurobiological findings as well as translational biomedicine studies that highlight working hypotheses, route(s) for SARS-CoV-2 entry into the CNS [5], relative consequences, post-infective neuropathology, potential relationships with neurodegenerative diseases [6], and therapy.

This Special Issue was launched at the beginning of the individuation of neuroCOVID-19 as a scientific and clinical field. The contributions of exceptional authors from around the world have stimulated various working hypotheses. As mentioned previously, in this editorial we highlight some progress in research on neuroCOVID-19, with the aim with the aim to depict new and more interesting experimental works, suggestions, opinions, and reviews capable of revealing unanswered questions about the mechanisms of neuroCOVID. Aimed at arousing the reader’s interest, this Issue showcases recent works that lead to conclusive considerations and open questions. More recently, an increased prevalence of new-onset autoantibodies after severe clinical manifestations of COVID-19 was found, suggesting its close association with neuropsychiatric symptoms of post-COVID-19 [7]. The potential link between specific autoantibodies and the neurological symptoms of post-COVID-19 syndrome was hypothesized as a biological explanation to explain the persistence of muscular and neurological clinical manifestation two to three years after Sars-CoV-2 infection. On the other hand, radiological studies have looked for neuroanatomical and structural changes in the structures of the central and peripheral nervous system. Scholars who consider potential brain structural changes over time, far from the pandemic event, are highlighting microstructural alterations of cortical and subcortical areas. By quantitative susceptibility mapping (QSM), a recently developed magnetic resonance imaging (MRI) technique for quantifying the spatial distribution of magnetic susceptibility within biological tissues, an increase in susceptibility to MRI in the medulla, pons, and midbrain regions of the brainstem of post-COVID-19 patients was found. In these regions, a greater tissue sensitivity corresponded with a more severe clinical picture, a greater expression of inflammatory biomarkers, and a significantly worse functional recovery [8]. Furthermore, it is important to highlight the use of neuroCOVID-19 databases as a source of biological, clinical, and therapeutic data of the neurological condition associated with COVID-19 disease. Launched at the beginning of the pandemic, data collection has made it possible to follow the evolution of the scientific research evidence produced in recent years [9,10]. This Special Issue attempted to highlight the neuroCOVID-19 aftermath and ongoing challenges. The researchers and clinicians who joined are allied in unraveling the molecular and cellular pathogenetic mechanisms and the clinical development of this unexpected SARS-CoV-2 infectious evolution, mapping the geography of neuroCOVID-19 [11,12].
